# Acute Effects of Particulate Matter on All-Cause Mortality in Urban, Rural, and Suburban Areas, Italy

**DOI:** 10.3390/ijerph182412895

**Published:** 2021-12-07

**Authors:** Matteo Renzi, Stefano Marchetti, Francesca de' Donato, Marilena Pappagallo, Matteo Scortichini, Marina Davoli, Luisa Frova, Paola Michelozzi, Massimo Stafoggia

**Affiliations:** 1 Department of Epidemiology, Lazio Region Health Service/ASL Roma 1, 00147 Rome, Italy; f.dedonato@deplazio.it (F.d.D.); m.scortichini@deplazio.it (M.S.); m.davoli@deplazio.it (M.D.); p.michelozzi@deplazio.it (P.M.); m.stafoggia@deplazio.it (M.S.); 2 Division of Integrated Systems for Health, Social Assistance and Welfare, Italian National Institute of Statistics, 00184 Rome, Italy; stmarche@istat.it (S.M.); pappagal@istat.it (M.P.); frova@istat.it (L.F.)

**Keywords:** air pollution, mortality, nationwide, urbanization, geographical differences, low concentrations

## Abstract

Background: Short-term exposure to particulate matter (PM) has been related to mortality worldwide. Most evidence comes from studies conducted in major cities, while little is known on the effects of low concentrations of PM and in less urbanized areas. We aim to investigate the relationship between PM and all-cause mortality at national level in Italy. Methods: Daily numbers of all-cause mortality were collected for all 8092 municipalities of Italy, from 2006 to 2015. A satellite-based spatiotemporal model was developed to estimate daily PM_10_ (inhalable particles) and PM_2.5_ (fine particles) concentrations at 1-km resolution. Multivariate Poisson regression models were fit to estimate the association between daily PM and mortality at province level, and then, results were pooled with a random-effects meta-analysis. Associations were estimated by combination of age and sex and degree of urbanization of the municipalities. Flexible functions were estimated to explore the shape of the associations at low PM concentrations. Results: We analyzed 5,884,900 deaths (40% among subjects older than 85 years, 60% occurring outside the main urban areas). National daily mean (interquartile range) PM_10_ and PM_2.5_ concentrations were 23 (14) μg/m^3^ and 15 (11) μg/m^3^, respectively. Relative increases of mortality per 10 μg/m^3^ variation in lag 0–5 (average of last six days since death) PM_10_ and PM_2.5_ were 1.47% (95% Confidence Intervals (CI): 1.15%, 1.79%) and 1.96% (1.33%, 2.59%), respectively. Associations were highest among elderly and women for PM_10_ only, similar between rural and urbanized areas, and were present even at low concentrations, e.g., below WHO guidelines. Conclusions: Air pollution was robustly associated with peaks in daily all-cause mortality in Italy, both in large cities and in less urbanized areas of Italy. Current WHO Air Quality Guidelines (2021) for PM_10_ and PM_2.5_ are not sufficient to protect public health.

## 1. Introduction

Ambient air pollution is one of the most important risk factors for the human health worldwide. The World Health Organization (WHO) estimated that air pollution causes 4.2 million of deaths each year [1], which accounts for 6% of total deaths worldwide [2]. Moreover, in the last Global Burden of Diseases (GBD) [3] report, it has been observed that death rates related to outdoor air pollution are highest among the elderly [4]. In Italy, more than 20% of the population is aged over 65 years [5], making the health burden associated to air pollution a priority on the public health agenda. This implies the need for adequate measures to reduce air pollution levels.

During the last decades, many epidemiological studies have been conducted to assess the association between short-term exposure to outdoor air pollution and daily mortality [6,7,8]. Most studies have focused on particulate matter (PM) with a diameter of less than 10 (PM_10_) or 2.5 (PM_2.5_) micron [9,10,11,12], and a recent multi-country multi-city studied reported percent increases in risk per 10-unit increase of pollutant about 0.44% and 0.55%, respectively [8]. A recent systematic review that included 196 quantitative studies showed the harmful effects of PM_10_ and PM_2.5_ on all-cause and cause-specific (cardiovascular and respiratory) mortality [13]. In Italy, the Epiair and Epiair2 studies included 10 and 25 Italian cities, respectively, and reported significant effects of both PM_10_ and PM_2.5_ on cause-specific mortality and disease-specific hospital admissions [9,14]. Similarly, the European MED-PARTICLES project, which comprised 11 cities from three Mediterranean countries (Greece, Italy and Spain) as well as eight Italian cities, reported similar findings [11,15]. In particular, the authors reported a pooled estimate of ~0.5% risk increases per 10-unit increase in PM_2.5_ and PM_10_, which was approximately constant in the eight Italian cities.

To date, most of the studies were conducted in urban settings due to the location of air pollution monitoring stations in cities and health data availability. In the last few years, satellite observations have been increasingly used as alternative air pollution exposure data, as they provide relevant information to supplement ground-level measurements and offer a complete spatial coverage. Machine-learning methodologies have been recently applied in Italy considering satellite data, dispersion models, and land-use terms to estimate daily concentration of PM_10_ (2006–2015) and PM_2.5_ (2013–2015) at 1 × 1-km spatial resolution [16,17].

Due to the lack of data, little is known on particulate matter-related health effects in rural and suburban areas. Only few studies have focused on the different effects in these settings. Recently, Italian rural areas were considered in a study on the short-term exposure to PM on cardiovascular hospital admissions [18]. The authors assessed the associations both in urban and rural areas to test potential different effects. They found no differences in effect estimates between urban and non-urban areas. Moreover, the study allowed a comparison in the health effects of air pollution in the different geographical areas in Italy, which are characterized by a north-to-south decreasing trend in exposure [16,17] and socio-economic differentials [19], as reported in the annual Italian report of the National Institute of Statistics (ISTAT). Finally, most of the evidence about the PM-related health effects in Italy involved only cities of northern Italy.

Estimates from different settings are important not only to have a more thorough and complete picture of the short-term effects of air pollution at population level across the entire geographical domain but also to improve the knowledge basis on low-level exposure effects. Recently, a U.S. study conducted on the Medicare population, a cohort of subjects aged 65 years and over enrolled in the U.S. health insurance system, showed a significant effect of PM_2.5_ on mortality also for exposure levels below the U.S. standard (35 μg/m^3^) [20,21] In Europe, similar results were provided by an Italian study conducted at national level, which showed a positive association between PM_2.5_ and cardiovascular hospitalizations for low concentrations [18]. Currently, the European Air Quality legislation has fixed the limit of daily concentration of PM_2.5_ to 15 μg/m^3^ and for PM_10_ to 45 μg/m^3^, assuming there are no health effects below these levels.

The main objective of this study is to provide new evidence on the differential effects of short-term exposure to PM_10_ and PM_2.5_ on all-cause mortality across the entire Italian domain and by urban, sub-urban, and rural administrative settings. A secondary aim is to estimate the shape of the exposure-response function with a focus on the low levels of PM. Finally, we aimed to evaluate individual characteristics (sex and age) as potential effect modifiers of the PM-mortality association.

## 2. Material and Methods

### 2.1. Study Area

Italy is located in Southern Europe; it extends over 301,340 km^2^ and is divided into 8092 municipalities grouped into 110 administrative provinces, with a total population of 60,483,973 inhabitants in 2017 and a median number of inhabitants per municipality equal to 2438 (interquartile range, IQR = 5084).

### 2.2. Environmental Data

For each day, PM values were extracted by satellite information and refined by random-forest models. Details are available here [16,17]. Briefly, spatial and spatiotemporal predictors (e.g., aerosol optical depth (AOD), land use, and meteorological data) were obtained daily (2006–2015 for PM_10_ and 2013–2015 for PM_2.5_) and for each grid cell (1 × 1 km) of Italy. Models based on a four-stages approach using the spatial and spatiotemporal predictors were applied to predict daily mean values of PM_10_ and PM_2.5_ concentrations for each spatial unit (1 km^2^) of Italy. We limited the calculation for PM_2.5_ on the 2013–2015 period due to lack of monitoring data. Cross-validated R^2^ were 0.75 and 0.81 for PM_10_ and PM_2.5_, respectively.

We obtained daily values of air and dew point temperatures from the ERA-5 reanalysis model [22], released by the European Centre for Medium-Range Weather Forecasts (ECMWF). We collected data on air and dew point temperature estimated at 2-m height at 0.00 a.m. and 12.00 p.m. with a spatial resolution of 0.125° × 0.125° (approximately 10 × 10 km). We calculated daily mean values of air and dew point temperature by averaging the two daily retrievals. We finally calculated the daily apparent temperature using the formula (1).

AT = − 2.653 + (0.994 × Ta) + (0.0153 × Td^2^) (1)

where Ta is air temperature, and Td is dew point temperature. Apparent temperature (AT) is an index of human discomfort during hot and humid days, defined as a person’s perceived air temperature [23,24].

Lastly, we computed daily PM and apparent temperature for each municipality by averaging the grid-based estimates from the cells intersecting each spatial unit, with weights proportional to the intersection areas. 

### 2.3. Health Data

We collected information on deaths from the National Institute of Statistics (ISTAT). We computed daily counts of all-cause deaths for each municipality and also by sex and age group: 0–64, 65–74, 75–84, and 85+ years.

### 2.4. Statistical Analysis

We estimated the association between all-cause mortality and PM_10_ and PM_2.5_ by applying a pooled analysis by province. Italy is divided into 110 administrative provinces, which have on average 74 cities (range 6–315). Province-specific analyses were applied to better adjust for some confounders, such as time trends and temperature, but also for computational issues. In this way, we built 110 province-specific datasets based on daily- and city-specific observations. As a second step, we ran random-effects meta-analysis to pool the 110 province-specific estimates obtained in the first step.

We used the time-series study design with over-dispersed Poisson regression models to estimate the associations between daily exposure to PM at different time-windows (lag) and daily numbers of deaths. The associations were adjusted for time-trends, temperature, city indicator, national holidays, summer population decreases, and regional-specific influenza epidemics.

More details are provided in the Supplemental Data.

We defined three-time windows of PM exposure to evaluate the immediate (lag 0 and lag 0–1), delayed (lag 2–5), and prolonged effects (lag 0–5).

We tested for effect modification by combination of sex and age class (divided in eight classes: 0–64 males, 0–64 females, 65–74 males, 65–74 females, 75–84 males, 75–84 females, 85+ males, and 85+ females) by creating daily stratum-specific counts. We decided to analyze those potential effect modifiers together to better consider the different age composition between sexes. In addition, we evaluated a potential effect modification by degree of municipality urbanization using a stratified approach. We ran the analysis in a province- and urbanization level-specific datasets. We used the degree of urbanization (DEGURBA) classification with 3 levels of urbanization (urban, suburban, and rural), obtained by EUROSTAT [25]. In addition, as a sensitivity analysis, we also considered as an index of urbanization provided by ISTAT, which classifies each municipality by using socio-demographic characteristics, such as the average distance to local and health services, etc. (www.istat.it, (accessed on 1 April 2021)). More information is provided in the supplemental material.

We estimated exposure-response functions for each outcome by applying distributed-lag non-linear models (DLNM) in each municipality and pooling the resulting curves using a multivariate meta-regression [26] in order to capture the heterogeneity of estimates across space. Specifically, we modelled the function by using natural splines with 3 degrees of freedom. Exposure-response functions were extrapolated also by level of urbanization.

Finally, risk maps for both PM_10_ (2006–2015) and PM_2.5_ (2013–2015) are provided by region to explore the geographical differences. Regional estimates were obtained by meta-analysis of province-specific results for each Region.

Results are provided as the percent difference of mortality (with 95% confidence intervals (95%CI)) relative to 10 μg/m^3^ fixed increases in PM. We conducted all analyses with R (version 3.1.3; Institute for Statistics and Mathematics, WU Wien, Vienna, Austria).

## 3. Results

The average value of PM_10_ was 23.3 μg/m^3^ (standard deviation = 14.2) during the whole study period and 21.1 (13.6), and 15.1 (10.9) μg/m^3^ during the last three years of the study (2013–2015) for PM_10_ and PM_2.5_, respectively. The interquartile ranges (IQR), calculated as the difference between the 75th and 25th percentile, were 12.4 and 8.3 μg/m^3^ for PM_10_, and PM_2.5_ in the entire (2006–2015) and restricted (2013–2015) periods, respectively. Finally, mean values of air and apparent temperature were 11.8 °C and 10.9 °C, respectively (Table 1).

A total of 5,884,900 deaths for all causes were registered during the 2006–2015 period, while 1,839,300 were observed in the last three years (2013–2015) in Italy (Table 2). Overall, 48% of all deaths were among males, and 40% were among subjects aged 85 years and over. Urban and suburban areas accounted for 80% of total deaths, while only 20% were observed in rural areas.

**Figure 1:**
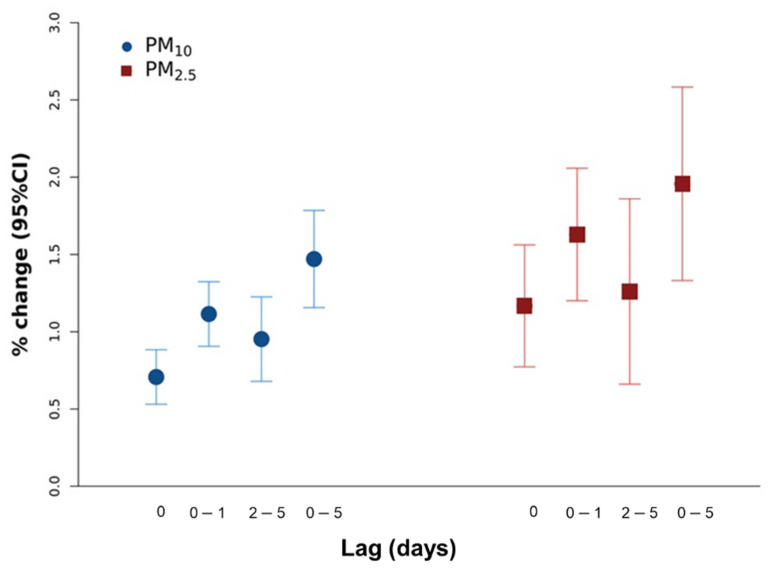
Associations between short-term exposure at different time windows (lag 0, 0–1, 2–5, 0–5) to PM_10_ and PM_2.5_ and all-cause mortality during 2006–2015 period for PM_10_ (blue dots) and 2013–2015 for PM_2.5_ (red dots). Results are from random effects meta-analysis of Italian province-specific estimates (110 provinces) and are expressed as percent change of risk and relative 95% confidence intervals per 10 μg/m^3^ increases.

Each 10-μg/m^3^ increase in PM_10_ was associated with statistically significant increments in mortality for each lag considered, with a percent change in risk up to 1.47 (95%CI: 1.16, 1.79) at lag 0–5 during the period 2006–2015. In the restricted period, similar positive associations for both PM_10_ and PM_2.5_ were observed with percent changes of 1.20% (95%CI: 0.84, 1.57) and 1.96% (95%CI: 1.33, 2.59), respectively, at lag 0–5 (Figure 1).

Table 3 shows effect modification by age and sex for PM_10_ (2006–2015) and PM_2.5_ (2013–2015). We observed an increasing trend in the risk of mortality by age in both sexes. The highest effects are among females aged 85 years and over for PM_2.5_ (3.07%: 95%CI: 2.07, 4.09) and among males aged 75–84 years (3.16%: 95%CI: 1.46, 4.89).

**Figure 2:**
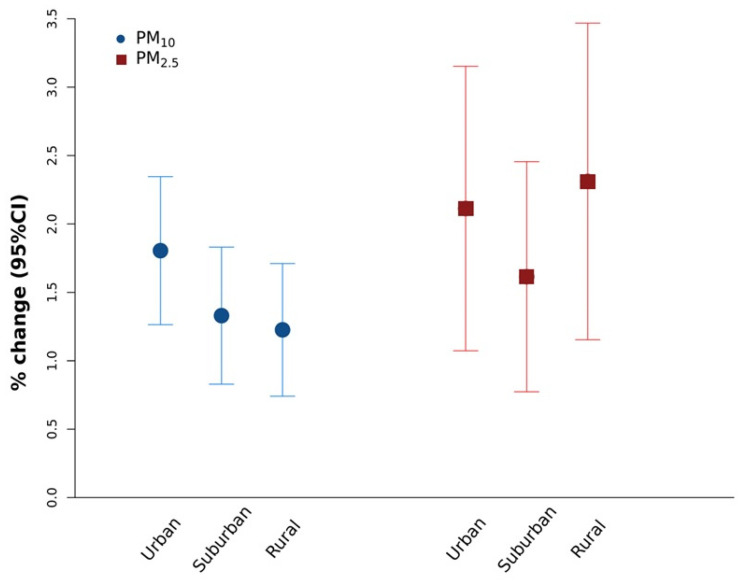
Effect modification for urbanization level (in 3 classes: rural, suburban, and urban cities) between PM_10_ and PM_2.5_ at lag 0–5 and all-cause mortality during 2006–2015 period for PM_10_ and 2013–2015 for and PM_2.5_. Results are expressed as percent change of risk and relative 95% confidence intervals per 10 μg/m^3^ increases.

When we evaluated urbanization as an effect modifier of the PM-mortality association, estimates were very similar, and we could not detect meaningful differences between rural, sub-urban, and urban areas (Figure 2), an important result demonstrating adverse effects of air pollution even outside major cities. 

**Figure 3:**
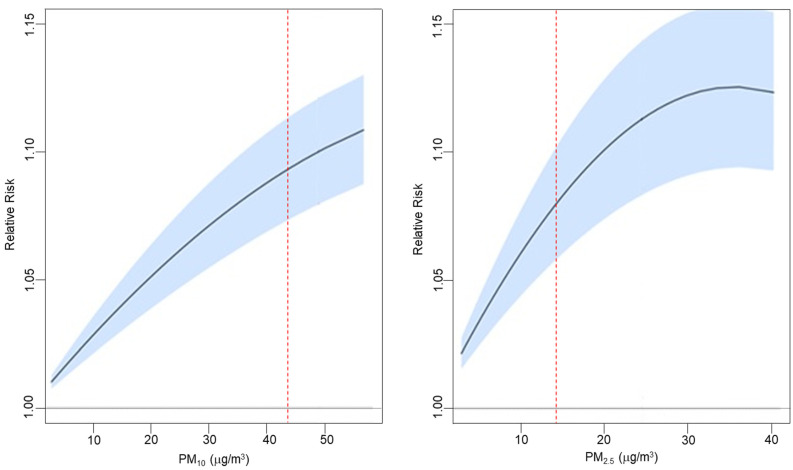
Exposure-response functions for PM_10_ (2006–2015) and PM_2.5_ (2013–2015) and all-cause mortality at lag 0–5 in Italy. Red line represents the meta-curve obtained by the 110 province-specific estimates. Red lines represent WHO guideline values for daily mean concentrations.

Figure 3 displays the pooled exposure-response functions for all-cause mortality at lag 0–5 for both PM_10_ and PM_2.5_. Both PM_10_ and PM_2.5_ exposure-responses curves were almost linear. The PM_2.5_ curve shows a steady increase in effect estimates for increases in PM, and for higher concentrations (35–40 μg/m^3^), it reaches a plateau when effects seem to remain constant thereafter. The PM_10_ curve is consistent with a linear trend. For both PM exposures, we found increased mortality risk below the current WHO guideline values for daily means of 15 (PM_2.5_) and 45 (PM_10_) μg/m^3^.

**Figure 4:**
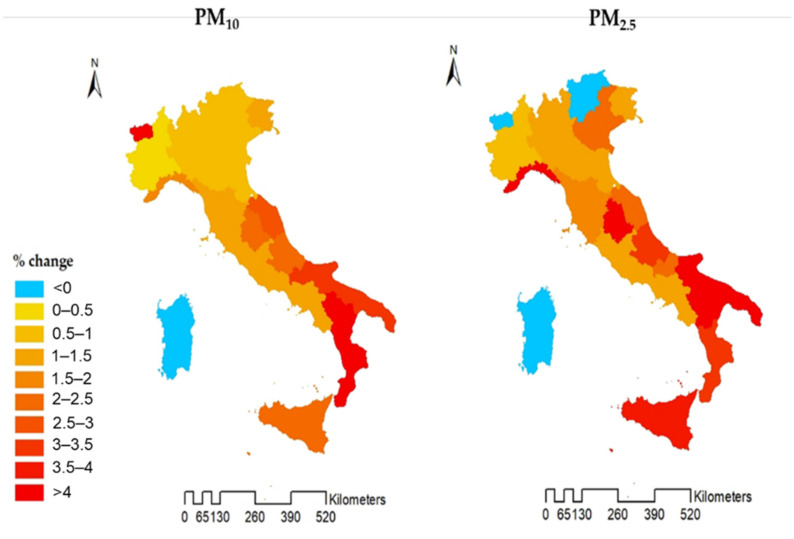
Association between PM_10_ and PM_2.5_ exposures at lag 0–5 and all-cause mortality in 20 Italian regions during 2006–2015 (PM_10_) and 2013–2015 (PM_2.5_) periods. Not statistically significant results are dulled. Results are expressed as % change of risk.

In Figure 4 region-specific effect estimates are reported for PM_10_ and PM_2.5_ at lag 0–5, respectively. Maps shows no clear geographic trend with positive associations in most regions; however, some regions in the south of Italy seem to have slightly higher effect estimates (Apulia, Sicily, Calabria). Some small mountainous regions (Valle d’Aosta, Trentino Alto Adige) or islands, such as Sardinia, showed no effect possibly due to sparse data. 

## 4. Discussion

In this paper, we investigated the short-term effects of PM_10_ and PM_2.5_ on all-cause mortality in the period 2006–2015 across the entire Italian domain using high-resolution exposure data and municipality-level mortality data. We found a positive association for both PM_10_ and PM_2.5_ and mortality with higher effects at the cumulative exposure level (lag 0–5 days). Significant effects were observed for both PM_10_ and PM_2.5_, starting form very low concentrations below the current EU air quality standards and WHO guidelines. We found a greater risk among the elderly and comparable effects in rural, suburban, and urban settings.

The association between short-term exposure to PM_10_ and PM_2.5_ and mortality has been well documented in the epidemiological literature. Recently, a systematic review collected information from 196 quantitative studies [13]. Specifically, the authors reported 66 studies on the acute exposure to PM_10_ and all-cause mortality and 29 studies focusing on PM_2.5_-related effects. In both cases, they found a positive association between PM and mortality, with percent changes in risk of 0.4 (80% prediction intervals: 0.3, 0.5%) and 0.7 (80%PI: 0.4, 0.9%) for a 10-μg/m^3^ increase in PM. In our studies, risk estimates were much higher for both PM_10_ and PM_2.5_. These differences might be due to a diverse setting (nationwide vs. multi-cities) considered in the studies. For example, in the multi-cities studies, only urban areas were considered, while in nationwide studies like ours, the heterogeneity is greater due to the diversity of settings considered both in terms of population size and characteristics, level of urbanization, and geographical context, which is characterized by different areas with higher heterogeneity. Previous estimates for Italy were provided by the Epiair multicity study [9]. Findings from the mortality study on 25 Italian cities in the period 2006–2010 showed a mortality risks of 0.51% (0.16%, 0.86%) and 0.78% (0.12%, 1.46%), respectively, for a 10-μg/m^3^ increase in PM_10_ and PM_2.5_. Worldwide similar estimates were observed in the recent multi-country study conducted in 652 cities by the Multi-City Multi-Country (MCC) Collaborative Research Network [8]. Higher effects of PM_2.5_ compared to PM_10_ have been commonly found in the epidemiological literature [9,27]. These results may be supported by the abundant evidence that this particulate fraction contains far more smaller particles that can absorb toxic components from the air and penetrate deep into the lungs [8,28].

Biological mechanisms underlying the association between inhalation of PM and health outcomes, such as mortality, have been widely investigated in the last years [12,29,30,31,32]. A recent clinical trial demonstrated that higher PM exposures can activate the human central nervous system, which induces the production of hormones as glucocorticoids, corticotropin-releasing hormone, and adrenocorticotropic hormone [32]. These processes are involved in physiological pathways for the increase in blood pressure and insulin-resistance, which are related to major health outcomes.

The joint interaction between age and sex showed an increasing trend in the mortality risk by age for both males and females. Moreover, we found different trends among the very old (>85 years) between sexes with higher PM_10_-related effects in females compared to males and the opposite for PM_2.5_. Some studies described higher effects in women among elderly subjects [33,34,35]. Hong et al. found that elderly women were most susceptible to the adverse effects of PM_10_ on the risk of acute mortality from stroke [35], while Bateson and Schwartz observed a higher risk of mortality for acute PM_10_ exposure among elderly women and men aged 65–75 years old [33], similar to our findings for Italy. When considering differential effects by age, it has been reported that some inflammatory markers, such as the C-reactive protein, increased with age [36], causing a worsening health status and higher frailty [37]. Differences between males and females in PM-related health effects might be related to physiological (smaller airways, greater airway reactivity, and greater deposition of PM_2.5_ [38]), hormonal, biological, structural, and morphological differences in the two sexes [39].

Given the high-resolution dataset and national coverage, we were able to estimate PM-related mortality risks in urban and sub-urban and rural settings, for which, to date, there is limited evidence. It is worth mentioning that, in less urbanized municipalities, access to health care and services is limited, the population is generally older, with potentially less economical resources and characterized by worse lifestyle habits, such as smoking and diet (www.istat.it, (accessed on 1 April 2021)). All these factors may render the population less resourceful, with a worse general health status, and air pollution might act as an easy trigger for mortality.

The exposure response functions showed a linear or quasi-linear association between PM exposure and mortality as previously documented [40,41], with an increase in the risk even for low PM concentrations well below the EU limits and WHO guidelines. These results confirm some recent epidemiological findings; Hanigan et al. investigated the role of long-term exposure to air pollution on all-cause mortality in a Australian cohort [42]. The authors found that each 1-unit increase in PM_2.5_ exposure led to a 5% (−2, 12%) increase in the risk of dying, even for low average exposure levels of 4.5 μg/m^3^ PM_2.5_. Despite that the study focused on the long-term effects of PM, which display different mechanisms than short-term associations, the evidence showed the harmful role of air pollution even at low concentrations. Similarly, a study conducted in the U.S. on a cohort of elderly Medicare patients found a 1.61% (1.48, 1.74%) increase in the risk of mortality for a 10-unit change in PM_2.5_ when considering days with PM below the daily National Ambient Air Quality Standards (NAAQS) limit of 35 μg/m^3^ [20]. Current daily PM_10_ and PM_2.5_ air quality standards should be amended to account for more recent findings on the health effects at lower levels. 

Finally, we explored the spatial heterogeneity of PM-related health effects across the 20 Italian Regions. We did not observe considerable differences in the risk across regions in Italy, while some consistency was observed in effect estimates in the southern regions of Italy, with slightly higher values. Moreover, it is worth mentioning there are mortality inequalities in Italy, with a typical north-south gradient related to socio-economic and educational level differences [43]. However, this is only a speculative hypothesis because we do not have information on educational level or socio-economic factors that might act as an effect modifier to address this. It will be a matter for further studies in the field in Italy.

This study has some limitations that should be addressed. Firstly, we only had total mortality and were not able to disaggregate mortality by cause or distinguish accidental and natural causes, and this might introduce a slight misclassification bias. Secondly, although our exposure allowed for a national spatial coverage with high spatial resolution compared to point-monitoring data, we still considered daily average exposure at municipality level derived by 1-km^2^ daily estimations, which can introduce a Berkson’s type bias. Finally, the lack of individual information, such as clinical history or individual characteristics and behaviors, did not allow to evaluate effect modification for such covariates.

## 5. Conclusions

The study provides evidence of harmful effects of both PM_10_ and PM_2.5_ on all-cause mortality in urban, rural, and sub-urban settings in Italy from very low PM concentrations. Greater effects were observed among the elderly. Results seem to confirm that not only urban settings are exposed to harmful pollutants and that local policies and awareness campaigns regarding the PM-related health effects should be extended to sub-urban and rural settings.

## Figures and Tables

**Table 1:** Description of the environmental data for the study period 2006–2015, overall and by level of urbanization score (in bold) in Italy. Data are reported for the long period (2006–2015) regarding PM_10_ and temperature for the restricted period (2013–2015) regarding PM_2.5_.

Variable					Percentiles				
Overall		Mean	SD	Min	25th	50th	75th	Max	IQR
PM_10_	μg/m^3^	23.3	14.2	1.8	14.6	19.7	27.0	290.2	12.4
PM_2.5_	μg/m^3^	15.1	10.9	1.3	8.7	11.6	16.9	163.4	8.3
Temperature	°C	11.8	8.0	−23.2	6.0	12.0	17.8	35.8	11.8
**Rural areas**									
PM_10_	μg/m^3^	20.8 *	12	1.8	13.6	18.0	24.0	275.7	9.4
PM_2.5_	μg/m^3^	13.5 *	9.4	1.6	8.2	10.7	15.1	157.0	6.9
**Suburban areas**									
PM_10_	μg/m^3^	28.0 *	16	2.1	17.8	23.6	32.5	290.2	14.7
PM_2.5_	μg/m^3^	18.0 *	12.6	1.3	10.1	13.8	21.0	163.4	10.9
**Urban areas**									
PM_10_	μg/m^3^	34.1 *	21	2.7	21.4	28.0	39.0	283.6	17.6
PM_2.5_	μg/m^3^	21.3 *	15.6	1.3	11.3	15.6	25.1	158.3	13.8

* *t*-test *p*-value < 0.05.

**Table 2:** Description of the health data by individual characteristics (in bold) for the study period 2006–2015 overall and by level of urbanization score in Italy.

	Urban		Suburban		Rural		Overall
**Total**	N	%	N	%	N	%	N
	2,323,100	39.5	2,370,200	40.3	1,191,700	20.3	5,884,900
**Age 0**–**64**							
	358,660	47.2	274,660	36.1	127,230	16.7	760,550
**Age 65**–**74**							
	384,380	43.4	348,830	39.4	151,540	17.1	884,750
**Age 75**–**84**							
	746,640	39.3	779,680	41.0	374,070	19.7	1,900,400
**Age 85**+							
	833,280	35.6	966,930	41.3	538,830	23.0	2,339,000
**Males**							
	1,143,800	40.0	1,144,400	40.0	570,930	20.0	2,859,100
**Females**							
	1,179,300	39.0	1,225,700	40.5	620,780	20.5	3,025,800

**Table 3:** Effect modification for age class and sex between PM_10_ and PM_2.5_ at lag 0–5 and all-cause mortality during 2006–2015 period for PM_10_ and 2013–2015 for and PM_2.5_. Results are expressed as percent change of risk and relative 95% confidence intervals per 10 μg/m^3^ increases.

Age Class	Sex	PM_10_			PM_2.5_		
		% Change	95% CI		% Change	95% CI	
0–64	Females	−0.33	−1.86	1.22	−0.21	−5.23	5.06
	Males	−0.18	−1.14	0.78	−1.17	−3.46	1.18
65–74	Females	0.34	−0.76	1.44	0.45	−2.20	3.18
	Males	0.27	−0.63	1.18	0.09	−1.76	1.98
75–84	Females	1.68	1.01	2.35	1.42	−0.59	3.48
	Males	1.53	0.90	2.17	3.16	1.46	4.89
85+	Females	2.73	2.21	3.26	3.07	2.07	4.09
	Males	1.74	0.84	2.64	3.10	1.21	5.02

## Data Availability

The data that support the findings of this study are available on request from the corresponding author (M.R.). The data are not publicly available due to privacy restriction, as they contain information that could compromise research participant privacy/consent.

## Supplementary Materials

The following are available online at https://www.mdpi.com/article/10.3390/ijerph182412895/s1.
