# Supplementary material for: Acute Effects of Particulate Matter on All-Cause Mortality in Urban, Rural, and Suburban Areas, Italy
Source: Int J Environ Res Public Health. 2021 Dec 7;18(24):12895. doi: 10.3390/ijerph182412895 (PMC8701500; doi:10.3390/ijerph182412895)
Supplement: Supplementary file 1 [file ijerph-18-12895-s001.zip › ijerph-1450366-Supplemental material.pdf]

Supplemental material

1. Statistical analysis – Confounders selection

We added in our models: long term and seasonal time trends, air/apparent temperature, municipality indicator, national holidays, summer population decreases, and region-specific influenza epidemics. Long-term and seasonal city-specific trends were modelled with natural splines with 6 degree of freedoms (df) per year. Air and apparent temperature were considered separately for warm (April to September) and cold (October to March) seasons. We used the city-specific median values of air temperature as a threshold to set warm or cold temperature. We adjusted for warm temperatures by modeling mean apparent temperature on the same day and previous day (lag 0–1). We have used natural splines with 2 knots, at 75th and 90th percentiles of the city-specific distribution, to model the warm temperatures. Similarly, we adjusted for cold temperatures by modeling the mean air temperature of the previous six days (lag 1–6) by natural splines with a single knot fixed on the 25th percentile of the city-specific distribution. Those variables were kept constant to/until the median value on days above/below for the warm and cold temperatures, respectively. Holidays were adjusted by a two levels indicator term (assigned a value of 1 on national and city holidays and 0 on other days), while summer population decreases by a three-levels variable (assigned a value of 2 for days during the 2-wk holiday period in mid-August, 1 for all other days during the period July 16–August 31, and 0 otherwise). Finally, influenza epidemics were added in the models by an indicator variable (coded as 1 for days during peak incidence periods, up to a maximum of 3 consecutive wk within each year, and 0 otherwise) with regional spatial resolution, because the data availability.
